# Host Transcription Factors in the Immediate Pro-Inflammatory Response to the Parasitic Mite *Psoroptes ovis*

**DOI:** 10.1371/journal.pone.0024402

**Published:** 2011-09-07

**Authors:** Stewart T. G. Burgess, Tom N. McNeilly, Craig A. Watkins, Alasdair J. Nisbet, John F. Huntley

**Affiliations:** Moredun Research Institute, Pentlands Science Park, Edinburgh, Scotland, United Kingdom; University of Michigan School of Medicine, United States of America

## Abstract

**Background:**

Sheep scab, caused by infestation with the ectoparasitic mite *Psoroptes ovis*, results in the rapid development of cutaneous inflammation and leads to the crusted skin lesions characteristic of the disease. We described previously the global host transcriptional response to infestation with *P. ovis*, elucidating elements of the inflammatory processes which lead to the development of a rapid and profound immune response. However, the mechanisms by which this response is instigated remain unclear. To identify novel methods of intervention a better understanding of the early events involved in triggering the immune response is essential. The objective of this study was to gain a clearer understanding of the mechanisms and signaling pathways involved in the instigation of the immediate pro-inflammatory response.

**Results:**

Through a combination of transcription factor binding site enrichment and pathway analysis we identified key roles for a number of transcription factors in the instigation of cutaneous inflammation. In particular, defined roles were elucidated for the transcription factors NF-kB and AP-1 in the orchestration of the early pro-inflammatory response, with these factors being implicated in the activation of a suite of inflammatory mediators.

**Conclusions:**

Interrogation of the host temporal response to *P. ovis* infestation has enabled the further identification of the mechanisms underlying the development of the immediate host pro-inflammatory response. This response involves key regulatory roles for the transcription factors NF-kB and AP-1. Pathway analysis demonstrated that the activation of these transcription factors may be triggered following a host LPS-type response, potentially involving TLR4-signalling and also lead to the intriguing possibility that this could be triggered by a *P. ovis* allergen.

## Introduction

Sheep scab, caused by the mite *Psoroptes ovis* is highly contagious, causing pruritis and irritation. It is a major welfare concern in livestock production in many areas of the world [1] and is currently the most important ectoparasitic disease of sheep in the UK. Current disease control strategies are reliant upon chemotherapy; however problems with residues, eco-toxicity and parasite resistance have raised concerns regarding such strategies [2]. Developing alternative control methods, for example vaccination or development of novel therapeutics, requires a deeper understanding of both the parasite and its interaction with the host.

Establishment of a *P. ovis* infestation is the result of a complex interaction between host and mite, during which the mite appears to initiate host reactions conducive to its own establishment and maintenance [3]. The life cycle of *P. ovis* is completed entirely upon the host [4] and mites survive on the surface of the skin, though their mouthparts do not appear to penetrate beyond the stratum corneum [5]. The available evidence suggests that mites abrade the stratum corneum, depositing allergens as they progress. This combination of skin abrasion, allergen deposition and self-grooming behaviour, initiated by the host in response to the pruritis caused by the mites, triggers the subsequent activation of a cutaneous inflammatory response [6] including an exudate which supplies the mite with a food source consisting of serous fluids, lymph and blood cells [7], [8]. Terminally differentiated keratinocyte cells within the stratum corneum, termed corneocytes, are therefore the first point of contact between the parasite and the host innate immune system.

A major feature of sheep scab is the rapid epidermal influx of eosinophils and neutrophils, followed by blister formation and a serous fluid exudate [9]. Increases in dermal mast cells occur by 96 hours post-infestation, and *P. ovis*-specific IgE is detectable within one week post-challenge [6]. These histopathological and serological changes suggest that an immediate-type hypersensitivity reaction is involved in lesion development [10]. However, this hypersensitivity reaction does not fully explain the initial pro-inflammatory response, as it can occur in mite-naïve sheep which lack *P. ovis*-specific IgE. The exact mechanism(s) by which the mite induces an early pro-inflammatory response, which represents a pivotal step in mite colonisation and is also critical in determining disease progression, are currently unknown.

We have previously described the global transcriptomic analysis of the host response to infestation with *P. ovis*, elucidating the inflammatory processes which lead to the development of a rapid and profound immune response [11]. This was achieved through microarray analysis of RNA extracted from sheep skin biopsy samples removed following infestation with *P. ovis*. Here we focus on the role of transcription factors in this process and use a combined clustering and pathway mapping approach to investigate the mechanisms by which the ubiquitous transcription factor nuclear factor-kB (NF-kB) specifically influences the host response to infestation.

## Results and Discussion

### Ethics Statement

Ethical approval for this study was obtained from the Moredun Research Institute.

Experiments Committee under experiment number: E19/08. Animals were monitored daily in accordance with guidelines agreed with the UK Home Office.

### Microarray study and data analysis

The experimental details of the microarray study for the analysis of the host response to skin infestation with *P. ovis* mites over a 24 hour time course have been described previously [11]. Briefly, *P. ovis* mites were applied to the skin of sheep (n = 6) and skin biopsy samples were removed following 1, 3, 6 and 24 hours of exposure along with reference (no infestation) biopsies. RNA was extracted from the skin biopsies and the transcriptional profiles were analysed using an ovine transcriptome microarray (Agilent, UK). This analysis identified 1,552 genes that were significantly differentially expressed at the transcript level in at least one of the 10 possible time point comparisons [non-infected control (C) *vs* 1 hour post infestation (hpi), C vs 3hpi, C vs 6hpi, C vs 24hpi, 1hpi vs 3hpi, 1hpi vs 6hpi, 1hpi vs 24hpi, 3hpi vs 6hpi, 3hpi vs 24hpi and 6hpi vs 24hpi]. Multiple test correction was performed using the Benjamini & Hochberg False Discovery Rate (FDR) procedure with an FDR corrected p-value cut-off set at ≤0.05, indicating that 5% of these genes could be expected to pass this filtering step by chance and may represent false positives (77 genes). Of the 1,552 probes, gene symbol level annotation was available for 1,383 probes (89%); the relevant homologous human gene symbol was used where the ovine annotation was unavailable. This annotation was obtained either from the Agilent ovine gene expression microarray annotation data or from individual BLAST analysis of the EST sequences represented by each probe, leaving 169 probes (11%) for which no annotation was available. These probes were excluded from the analysis described below, leaving 1,383 annotated probes available. Protocols of the experimental procedures, methods of analysis and microarray data are available as supplementary information in the European Bioinformatics Institute's ArrayExpress database (www.ebi.ac.uk/arrayexpress) accession number E-TABM-1012 and are fully compliant with the MIAME guidelines.

### Temporal modulation of the host inflammatory response

**Figure 1:**
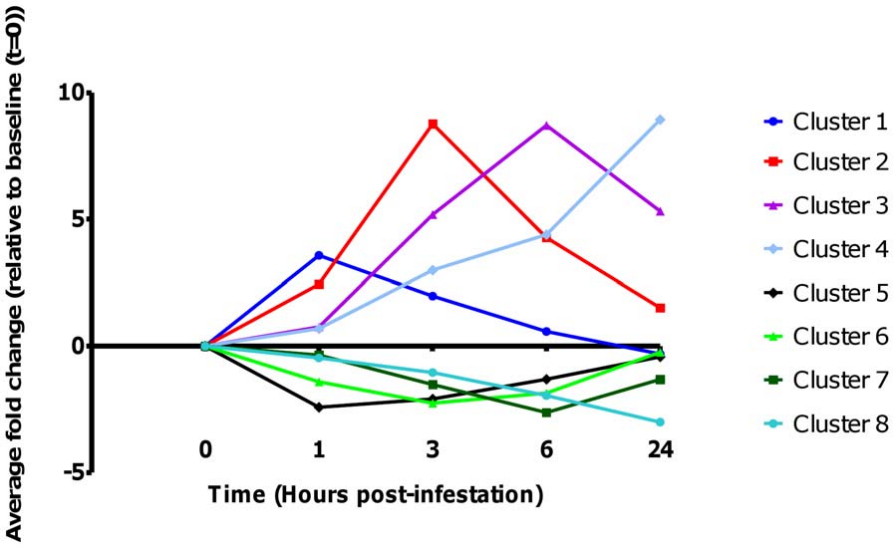
Clustered mean temporal gene expression profiles post-infestation with *P. ovis*. Chart shows the mean gene expression profiles for clusters 1–8. Time (hours) post infestation highlighted on X-axis (0, 1, 3, 6 and 24 hours) and mean fold change compared to time zero sample on the Y-axis.

All significantly differentially expressed genes were grouped into eight distinct clusters based on the time point (post-infestation) at which their expression level peaked. These profiles were calculated using the mean fold change profile of all genes in a cluster over the time course of infestation, as compared to the baseline (Time  = 0) sample data (Figure 1). For example genes in cluster 1 showed a peak of expression at 1 hpi and then quickly declined back to baseline levels (or below) by 6 hpi. Genes in cluster 2 showed increased expression until 3 hpi and then dropped back towards baseline levels by 24 hpi. The transcript level of genes in cluster 3 increased to a maximum at 6 hpi and then tapered off by 24 hpi, whilst transcripts representing genes in cluster 4 demonstrated a steady increase in expression over the 24 hour time course (Figure 1). Similar patterns were observed for the genes whose expression levels were suppressed following mite infestation (Figure 1). The process of clustering genes based on the time of their peak transcript expression level over the time course of infestation with *P. ovis* represented a successful method of grouping genes and allowed the investigation of the temporal patterns of gene expression in infested skin to be further analysed.

### Transcription factor binding site enrichment analysis via the bioinformatical analysis of human orthologous promoter sequences

In order to determine the role(s) of specific transcription factors in controlling the host temporal response to infestation the sequences of the promoter regions of the genes from each of the individual clusters (clusters 1–8) were obtained and analysed for the enrichment of specific transcription factor binding sites using the oPOSSUM Human Single Site Analysis package [12]. Due to the current absence of a complete, annotated ovine genome sequence, the human orthologous gene promoters were used to identify transcription factor binding motifs enriched within the gene clusters. Although there are likely to be distinct differences within the promoters of the selected genes between these two species, the levels of homology are such that this is considered to be a valid approach to this analysis [13]. In addition, by analysing clusters rather than focusing on individual genes we are able to limit the impact of any differences in binding motif distribution between species. Of the 1,383 annotated genes, human orthologous gene promoter information was available for 912. The results of a previous analysis suggested that the inducible transcription factor NF-kB plays a key role in the host response to infestation with *P. ovis* [11]. In the current analysis, potential NF-kB binding sites were found to be highly enriched in the promoter regions of genes within clusters 1, 2 and 3 (1, 3 and 6 hpi, respectively), indicating an activation role for NF-kB in orchestrating the early host response to infestation in ovine skin (Table 1).

**Table 1:** Enriched transcription factor binding motifs within temporal gene expression clusters following infestation with *P. ovis.*

Temporal cluster	Peak expression (hpi)	No. of genes in cluster	No of genes with mapped promoters	Over-represented transcription factor binding motifs in cluster (Z-score ≥5 or Fisher Score ≤ 0.05)
1	1	77	56	*RELA, MAX, NF-kB, REL, FOXD1, FOXQ1, LHX3, HLF, MYCN, CREB1, MEF2a, USF1, ARNT, GFI, FOXI1, SOX17, FOXA2, RORA1, SRF, TAL1-TCF3*
2	3	176	120	*REL, RELA, NF-KB, ELK1, SOX17, MZF1 (5*–*13), SP1, ELK4, SPIB, MZF1 (1*–*4), HNF4A, ELF5, FOS, CREB1, ZNF354C, SRY, NFKB1, ZEB1, USF1, ARNT-AHR*
3	6	316	201	*NF-kB, RELA, ESR1, PAX5, STAT1, ELF5*
4	24	361	229	*STAT1, TLX1-NFIC, SRF, GABPA, ELF5, IRF1, IRF2, SPIB*
5	−1	5	3	*E2F1*
6	−3	30	19	*ELK4, SRY, HLF, SOX5, FOXD1, FOXI1, SOX9, MYC-MAX, ROAZ, HNF1A, SPIB, SRF*
7	−6	237	163	*FOXF2, SP1, FOXQ1, FOXA2, LHX3, FOXD1, REL, NKX3-1, ARNT-AHR, MAX, CEBPA, NR3C1, IRF2, PBX1, FOXD3, BAPX1, IRF1, HLF, TEAD1, PRRX2*
8	−24	181	121	*HAND1-TCFE2A, PDX1, YY1, NOBOX, ZNF354C, SPIB, IRF1, SOX17, NKX2-5, MZF1 (1*–*4), ZEB1, MZF1 (5*–*13), DDIT3-CEBPA, LHX3, SP1, SOX5, ARNT, PRRX2, FOXF2*

### TF clusters with increased expression profiles

Within cluster 1 (peak expression at 1 hpi), 39 of the 56 genes with promoter information contained potential NF-kB binding sites. In addition, a number of binding motifs for other transcription factors were enriched within the promoter regions of genes in cluster 1, including those for serum response factor (*SRF*), hepatic leukaemia factor (*HLF*), RAR-related orphan receptor A (*RORA*) and cyclic AMP (CAMP) response element binding protein 1 (*CREB1*), which has defined roles in the instigation of host transcriptional response to inflammation [14]. The presence of *RORA* binding motifs is of interest as this transcription factor has been implicated in the control of epidermal differentiation [15]. *RORA* expression was down-regulated in our dataset with a peak repression of 2-fold observed by 24 hpi. Intriguingly the genes in cluster 1 showed a peak expression at 1 hpi which then quickly decreased with little or no expression of these genes observed by 24 hpi and this repression may be caused by a down-regulation of *RORA* at this time. Of the 120 genes with promoter information in cluster 2 (peak expression at 3 hpi), 71 had potential NF-kB binding sites, and motifs for the following transcription factors were also enriched; Spi-B transcription factor (*SPIB*) and *ELK1*. Of the 201 genes within cluster 3 (peak expression at 6 hpi) with promoter information available, 58 had potential NF-kB binding sites. In addition binding motifs were enriched for signal transducer and activator of transcription 1 (*STAT1*) and estrogen receptor 1 (*ESR1*). Cluster 4 (peak expression at 24 hpi) was enriched for binding motifs for *STAT1*, *ELF5*, *SPIB,* interferon response factor 1 (*IRF1*), and *IRF2*.

### TF clusters with decreased expression profiles

Cluster 5 (most repressed expression at 1 hpi) contained only five genes and promoter information was only available for 3 of these, 2 of which had binding site motifs for E2F transcription factor 1 (*E2F1*), which has roles in both cell proliferation and apoptosis [16]. Cluster 6 (most repressed expression at 3 hpi) showed enrichment for motifs for the forkhead box factors D1 (*FOXD1*) and I1 (*FOXI1*) and *SPIB*. Cluster 7 (most repressed expression at 6 hpi) also showed enrichment for a number of forkhead box factors with motifs for *FOXQ1*, *FOXA2*, *FOXD1*, *FOXD3* and *FOXF2*. Finally, cluster 8 (most repressed expression at 24 hpi) showed enrichment for binding sites for the following transcription factors, *IRF1*, LIM homeobox 3 (*LHX3*) and paired related homeobox 2 (*PRRX2*). Interestingly, *PRRX2* has defined roles in the development of the dermal layer and also in the cutaneous response to wounding [17]. The binding motifs for the transcriptional regulator *IRF1* were also enriched in both Cluster 4 (peak up-regulation at 24 hpi) and Cluster 8 (most repressed expression at 24 hpi), and therefore the final action of this transcription factor may be determined through its interactions with other transcriptional regulators, as previously demonstrated for IRFs ([18], [19]. The distinct lack of NF-kB binding motifs in the promoter regions of those genes with repressed expression profiles following infestation further highlights the pro-inflammatory nature of this transcription factor.

### Differential expression and temporal clustering of transcription factor genes following infestation with *P. ovis*

**Figure 2:**
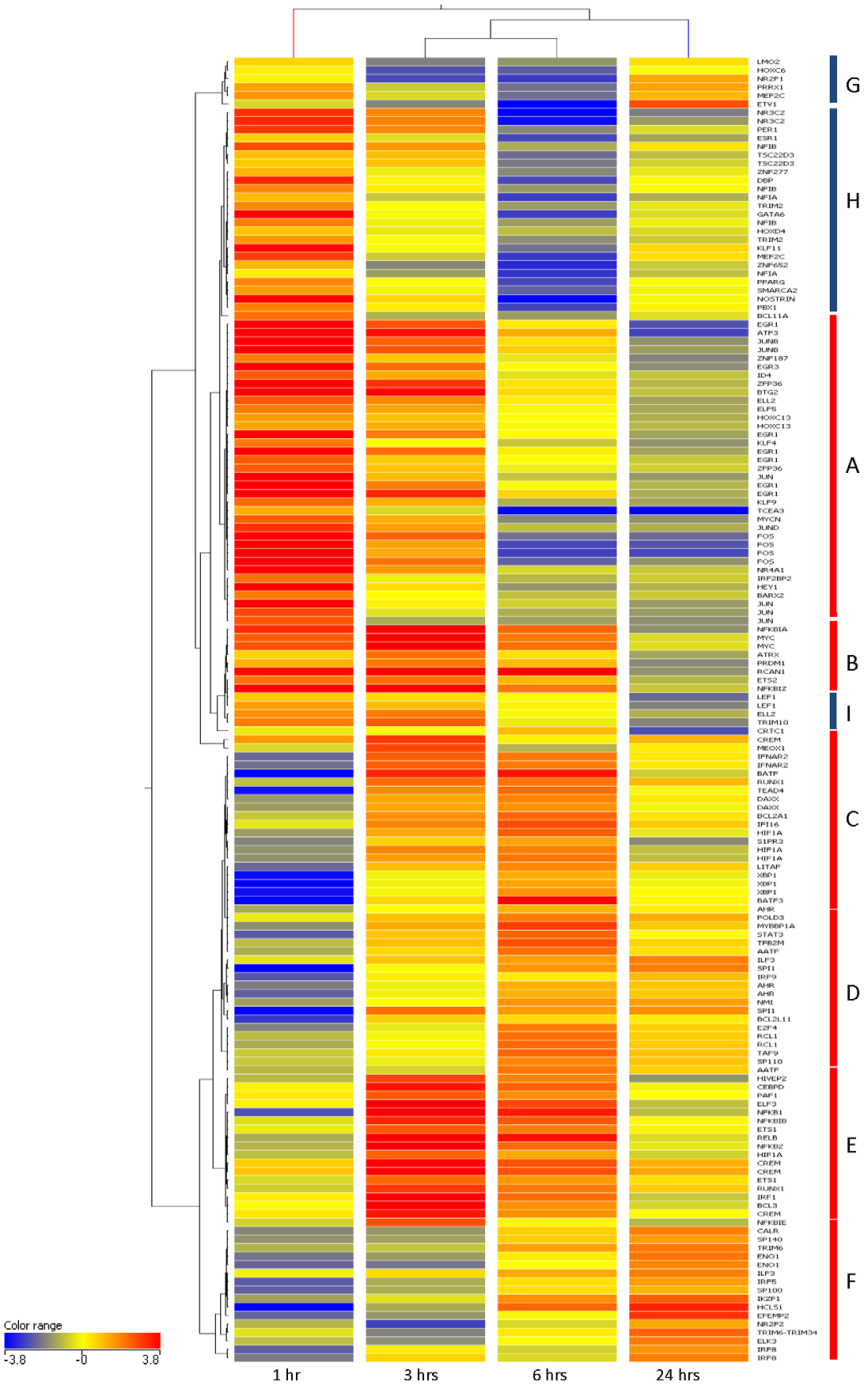
Hierarchical clustering analysis of transcription factor genes following infestation with *P. ovis*. Clustering preformed in Genespring GX 11.0 (Agilent, UK) using the Pearson Centered distance metric. Each block represents a single gene at each time point (Control (Time  = 0), 1 hour, 3 hours, 6 hours and 24 hours post-infestation). Genes are colour coded by log normalised intensity values. A-I  =  Clusters A –I (full description in text).

In order to define further the role of transcription factors in the host response to infestation we used the Ingenuity Pathway Analysis software to search for transcription factors amongst the genes differentially expressed across the time course. This search identified 151 genes (from the original list of 1,383 for which gene level annotation was available) with the functional annotation of either transcription factor or transcriptional regulator (∼11%) (File S1). Of these transcription factor genes the expression levels of 112 were increased following infestation, with 31 transcription factor genes showing peak expression of transcript at 1 hpi (no genes down-regulated), 33 at 3 hpi (3 genes down-regulated), 30 at 6 hpi (25 genes down-regulated) and 18 at 24 hpi (11 genes down-regulated). This demonstrates the temporal pattern of expression of transcription factor genes following infestation with sheep scab, with distinct sub-sets of transcription factors having increased expression early at 1, 3, 6 and 24 hpi, accompanied by down-regulation of specific transcription factors between 6 and 24 hpi. To characterise this response further we clustered transcription factors genes based on their expression profile over the time course of infestation with *P. ovis*. Hierarchical clustering of these genes was performed in Genespring GX (Agilent, UK) using the Pearson Centered distance metric (Figure 2). The early response to infestation with *P. ovis* (1–3 hpi) appeared to be dominated by the increased expression of a number of transcription factors involved in the initiation of the response to bacterial lipopolysaccharide (LPS) [20]. This response is highlighted by Cluster A (Figure 2), which shows the significantly increased expression of a number of Immediate Early Genes (IEGs), namely the transcription factors *JUN, JUNB, JUND, FOS, NR4A1, ATF3, DUSP1, EGR1* and *EGR3*. The transcription factors AP-1, of which *JUN, JUNB, JUND, FOS* and *ATF3* are members [triggered by mitogen activated protein kinase (MAPK) cascades)] and NF-kB are class I regulators of the response to LPS [21] and these genes are constitutively expressed, being present in latent form in the cell and becoming activated following specific signaling events (i.e. LPS binding to the TLR4 signaling complex) before going on to activate transcription of the primary response genes [20]. Increased expression of these IEGs at this time may be indicative of a positive feedback mechanism, whereby these genes not only instigate the transcription of the primary response to LPS but also go on to regulate their own expression ensuring their own replenishment for further cascades of transcription [21] **.** Interestingly Vroling *et al*., [22] defined similar roles for NF-kB and AP-1 in the response of primary nasal epithelial cells exposed to house dust mite allergens, indicating a conserved response to exposure to the allergens of two different mite species at two distinct sites of infestation, i.e. skin and nasal epithelium. A number of other transcription factors, namely *CEBPD, CREM, HIF1A, RUNX1, IRF1, BCL3, XBP1, BATF* and *BATF3* showed peak expression between 3 and 6 hpi (Clusters C & E, respectively). The *de novo* synthesis of these transcription factors is known to be induced during the primary response to LPS and they are then involved in the instigation of subsequent waves of gene expression following on from the primary response genes (i.e. the secondary response). A number of these factors have been described as class II regulators of the response to LPS [21]. A final group of transcription factors with peak expression at 24 hpi (*SPI1, AATF, STAT3, IRF5, IRF8* and *IRF9*) can be observed in Clusters D & F (Figure 2). Amongst these genes, *IRF8* and *SPI1* (*PU.1*) have been shown to play important roles in the activation and differentiation of macrophages, thus determining macrophage-specific patterns of inducible gene expression [23], [24]. The transcription factor *RUNX1* showed peak expression between 3 and 6 hpi and has been implicated in the control of regulatory T-cells through its interaction with another transcription factor *FOXP3*. This may be an important observation from a sheep scab perspective as T-regulatory cells have been shown to be present in sheep scab lesional skin [25], [26]. ATF3 functions as a negative regulator of toll-like receptor 4 (TLR4), via a negative feedback mechanism, and, in addition it can function as a transcriptional activator when coupled with JUN family members [27], [28]. In fact NF-kB, CEBPD and ATF3 have been shown to act in unison, forming a regulatory circuit, capable of discriminating between transient and persistent TLR4 mediated signals [28], [29]. NF-kB triggers weak transcription of *IL6*, at the same time that it regulates *CEBPD* which binds to the *IL6* promoter together with NF-kB stimulating maximum transcription of *IL6* [29]. These binding events are followed by the binding of ATF3 which acts to attenuate transcription of both *IL6* and *CEBPD*, thus inhibiting further IL6 transcription [29]. These findings demonstrate the complex and multi-factorial nature of the transcriptional control of the inflammatory response, highlighting that a successful and appropriate response can only be triggered through the coordinated action of multiple transcription factors acting through temporally controlled cascades of transcription.

A number of additional temporally regulated transcription factors can be observed from the hierarchical clustering analysis; Cluster B contains a number of genes whose representative transcripts were up-regulated at 1, 3 and 6 hpi (Figure 2), including two NF-kB inhibitory genes, *NFKBIA, NFKBIZ* along with *ETS2* and *MYC*. Cluster E (peak expression at 3–6 hpi) contains additional NF-kB inhibitory factors (*NFKBIB* and *NFKBIE*) along with genes encoding NF-kB sub-units (*RELB, NFKB1* and *NFKB2*). NF-kB is well characterised as activating the transcription of the genes which encode itself whilst at the same time activating the transcription of its own inhibitor proteins, the IkB factors, i.e. *NFKBIA, NFKBIB*, *NFKBIZ* and *NFKBIE* as part of a negative feedback mechanism [30]. These proteins are able to inactivate NF-kB by trapping it within the cytoplasm, thus preventing its nuclear translocation [30]. Unlike the other IkB proteins, NFKBIZ is able to inhibit NF-kB activity without affecting its nuclear translocation, instead it inhibits the DNA-binding of NF-kB [31]. NFKBIZ is also known to activate *IL6* transcription whilst decreasing transcription of *TNF* in response to LPS [32] and may also be involved in the induction of inflammatory genes activated through the TLR/IL-1 signaling pathways [33]. NFKBIZ has also been described as an essential molecule in the homeostasis of skin immunity, with targeted disruption of this factor leading to a severe atopic dermatitis-like disease in mice [34]; therefore correct regulation of NF-kB signaling may be a critical stage in determining an appropriate inflammatory response. Cluster H contains a number of genes that are up-regulated early (1 hpi) and then have lower transcription levels by 6 hpi, including *NFIA, NFIB* and *PPARG*, whilst Cluster G shows a number of genes with most repressed expression at 3 hpi (*LMO2, HOXC6, NR2F1* and *PRRX1*). Activation of *PPARG* has been implicated in the stimulation of keratinocyte differentiation and in the limitation of cutaneous inflammation [35]. Temporal analysis of transcription factor gene expression has therefore demonstrated a multi-factorial staged response following infestation with *P. ovis*. Examination of the factors involved during this response revealed the presence of an LPS-type reaction to infestation with *P. ovis*, likely to be modulated via the NF-kB and AP-1 signaling pathways and potentially leading to the instigation of the host pro-inflammatory response.

### NF-kB plays a defined role in the instigation of the host pro-inflammatory response following infestation of ovine skin with *P. ovis*

**Figure 3:**
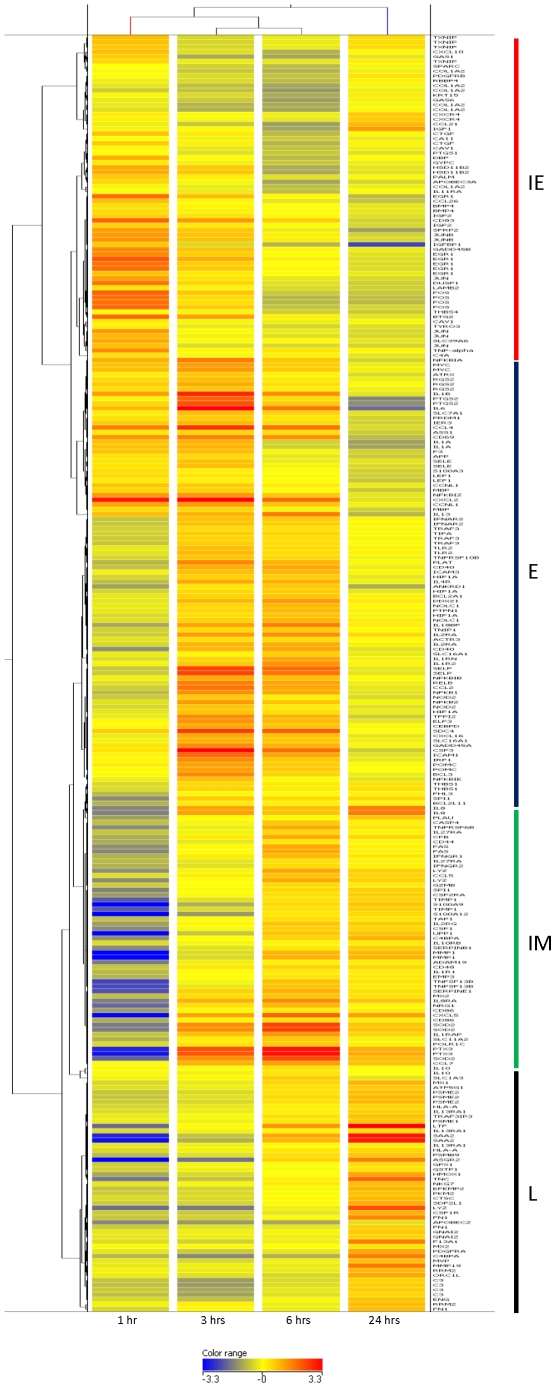
Hierarchical clustering of temporal expression of known and predicted NF-kB regulated genes following infection with *P. ovis*. Clustering preformed in Genespring GX 11.0 (Agilent, UK) using the Pearson Centered distance metric. Each block represents a single gene at each time point (Control (Time  = 0), 1 hour, 3 hours, 6 hours and 24 hours post-infestation). Genes are colour coded by log normalised intensity values. IE  =  Immediate Early gene cluster, E  =  Early gene cluster, IM  =  Intermediate gene cluster, L  =  Late gene cluster.

Promoter analysis highlighted the influence of NF-kB in the control of the transcriptional response to infestation with *P. ovis*; as such we focused on NF-kB, searching in greater detail across our dataset for genes with known or predicted NF-kB binding sites. Schreiber *et al*., [36] identified 419 genes whose promoters have been characterised as having at least one functional NF-kB binding site. To further supplement this list we obtained additional genes whose promoters are either characterised as being bound by NF-kB or that contain consensus binding sites for the transcription factor. These were obtained from the online resource NF-kB.org (http://www.nf-kb.org) and also from studies by Tian *et al*., [37] and Schreiber *et al*., [36], providing a combined total of 799 known and predicted NF-kB binding genes (File S2). The time course gene list was compared against this combined NF-kB list and of the 1,383 differentially expressed genes with available annotation, 259 were represented in the list of genes with either functional or predicted NF-kB sites in their promoters (19%) (File S3). This indicates that there is a core set of genes that are regulated by NF-kB following infestation with *P. ovis*, the vast majority of which were up-regulated with infestation (212/259 or 82%), with the percentage of NF-kB genes up-regulated in each of the temporal clusters as follows: cluster 1 (32%, 1 hpi), 2 (30%, 3 hpi), 3 (18%, 6 hpi) and 4 (17%, 24 hpi), indicating an early NF-kB dominated response that declines by 24 hpi. This supports our finding that NF-kB appeared to be involved early in the host response to infestation with *P. ovis* and it is likely that this transcription factor is activated during the initial contact between mite antigens and host keratinocytes [11]. Figure 3 shows the hierarchical clustering of the 259 potentially NF-kB regulated genes shown to be differentially expressed with time following infestation with *P. ovis*. This clustering clearly highlights temporal waves of NF-kB regulated gene expression, with an immediate early (IE) group of genes with peak expression at 1 hpi, an early (E) group of genes peaking at 3 hpi, an intermediate (IM) group peaking at 6 hpi and a late (L) group of genes peaking at 24 hpi (Figure 3). As described earlier the IE group contains a number of factors involved in the primary response to LPS, i.e. AP-1 family members, *TNF, EGR1* and *DUSP1*. However, the clustering of NF-kB regulated genes also revealed the peak expression of the eosinophil chemotactic cytokine *CCL26* at this stage. Eosinophilia is a characteristic feature of sheep scab pathogenesis with early lesions being dominated by rapid eosinophil infiltration, certainly occurring within the first 24 hours, however skin inflammation can be observed within 1 hour of exposure to *P. ovis* [6], [11]. The possibility that this influx could be, at least partly controlled by NF-kB could lead to the identification of novel methods of intervening in disease progression by targeting the pro-inflammatory response upon which the mites appear to thrive [38]. Perhaps unsurprisingly, the top biological function represented in the early (E) cluster (peak expression 3 hpi) of NF-kB regulated genes was “Inflammatory Disease” with 36 of the 175 genes available for pathway mapping being represented by this function (p-value range  = 6.5E^−25^-2.3E^−6^). This cascade of genes represents the peak expression of a number of pro-inflammatory factors, i.e. *IL1A, IL1B, CCL2, CCL4* and *IL6* and also those involved in the activation, binding and translocation of immune cells across the epithelia, i.e. *ICAM1, ICAM3, SELE* and *SELP* [39]. An additional factor with peak expression at this time was *PTGS2* [or cyclooxygenase-2 (COX-2)], which is an inducible factor and a major mediator of inflammation and prostanoid biosynthesis and signaling [40]. *PTGS2* is regulated by NF-kB in response to LPS exposure [41] and this pathway can be inhibited through the action of the non-steroidal anti-inflammatory drugs (NSAIDs) [42]. Therefore the inhibition of this pathway may represent an alternative means of intervention by disrupting the development of the pro-inflammatory response following exposure to *P. ovis*. *PTGS1* (COX-1) expression peaked at 1 hpi. The intermediate cluster of NF-kB regulated genes (peak expression at 6 hpi) is characterised by the presence of a number of immune regulators, including *IL8* which is one of the major mediators of inflammation, functioning as a potent chemoattractant factor [43]. Additional factors within the same cluster include the pro-inflammatory S100 genes *S100A9* and *S100A12* [44], [45]. *S100A9* is involved in the cellular response to LPS exposure and also controls up-regulation of *IL8* expression and increased surface expression of the adhesion molecule *ICAM1*, both of which are implicated in sheep scab pathogenesis [11], [46]. Both *S100A9* and *S100A12* are members of the epidermal differentiation complex (EDC) of genes which act to control terminal differentiation of keratinocytes. A number of additional EDC genes were identified in the original dataset, i.e. loricrin (*LOR*), filaggrin (*FLG*) and late cornified envelope 1B (*LCE1B*) were down-regulated whilst small proline rich proteins 2A (*SPRR2A*) and 2E (*SPRR2E*) and a number of S100 genes were up-regulated [11]. In fact transcription factors identified in this study, including NF-kB, AP-1, *ETS1*, *KLF4* and *MYC* have been implicated in the control of epidermal differentiation [47], [48], [49], [50], [51], [52]. Of particular interest is the 3-fold up-regulation of the transcription factor *ETS1* by 3 hpi. This factor has been implicated in the control of epidermal differentiation through its regulation of a number of EDC genes [53]. Over expression of *ETS1* in mice led to decreased expression of the EDC genes *LOR*, *FLG* and involucrin (*IVL*), leading to disrupted barrier function [53]. Therefore increased expression of the transcription factors NF-kB, *ETS1* and AP-1 following infestation with *P. ovis* may play a role in the regulation of a number of EDC genes, with potential effects on barrier function.

The clusters representing potential NF-kB-regulated genes with peak expression between 6 and 24 hours contain a number of factors involved in the acute phase protein (APP) response, i.e. pentraxin 3 (*PTX3*), serum amyloid A2 (*SAA2*), complement factor 3 (*C3*), fibronectin 1 (*FN1*) and superoxide dismutase 2 (*SOD2*). In particular *PTX3* is of interest as it is produced locally following primary pro-inflammatory signals (i.e. TLR activation) and acts to limit inflammation-mediated tissue damage by regulating apoptotic cell clearance and also regulates the classical complement pathway by binding complement component 1Q (*C1Q*) [54]. The transcription factor signal transducer and activator of transcription 3 (*STAT3*), also known as acute phase response factor (*APRF*) showed peak expression at 6 hpi and along with NF-kB this factor has been shown to be involved in the transcriptional activation of a range of APPs [55]. A number of factors involved in the APP response were up-regulated in the original dataset and these include haptoglobin (Hp), coagulation factors 3 (*F3*), 5 (*F5*) and 13A1 (*F13A1*) [11]. The APP response is a systemic response to local inflammation during which a number of APPs are rapidly produced by hepatocytes following activation by interleukins, i.e. IL1, IL6, IL8 and TNF-alpha, all of which are up-regulated in the skin following exposure to *P. ovis* [56]. APPs can also be expressed locally in response to inflammation and *HP* expression has been observed in keratinocytes following inflammatory cytokine signaling [57]. It is therefore likely that the APPs are released in response to the cutaneous inflammatory response induced by exposure to *P. ovis* in order to further regulate the developing immune response.

### The activation of NF-kB, TLR4-signaling and a mite-derived factor

The cascade of NF-kB regulated genes detailed above highlights the influence that this and other transcription factors have on the development of a cutaneous inflammatory response to *P. ovis*. This response ranges from the co-ordinated release of pro-inflammatory cytokines to produce a cytokine gradient, through which immune-mediator cells are attracted, activated and guided towards the site of infestation, to the expression of a range of integrins and adherins which facilitate this process. The triggering of this rapid and extensive inflammatory response by mite-derived factors is clearly of central interest in elucidating the initiation of the immunopathology associated with sheep scab. A previous study by our group demonstrated that expression of key members of the TLR4-signalling pathway including TLR4, MYD88 and CD14 where all up-regulated in sheep skin within a few hours of exposure to *P. ovis*, thus implicating an LPS-type response resulting in the activation of NF-kB and AP-1 [11]. The triggering of innate immune receptors by parasites is not novel, for example the parasitic nematode *Acanthocheilonema viteae* (via the ES62 molecule) and the parasitic trematode *Schistosoma mansoni* [via lacto-N-fucopentaose III (LNFPIII)] have been shown to induce NF-kB-mediated immune responses by activating the TLR4-signalling pathway [58], [59]. The identities of the factor(s) which trigger an LPS-type response in sheep scab are as yet unknown. However, there is evidence indicating that the house dust mite (HDM) allergen, Der p 2, can act as a functional mimic of myeloid differentiation protein -2 (MD-2) [60]. MD-2 is the LPS-binding component of the TLR4 signaling complex and enhances TLR4-dependent activation of NF-kB following binding of the TLR4 ligand LPS [61]. Trompette *et al*., [60] demonstrated that Der p 2 is able to facilitate signaling through direct interactions with the TLR4 complex, and could reconstitute LPS-driven TLR4 signaling even in the absence of MD-2. Der p 2 was also shown to activate dendritic cells and induce a Th2-biased immune response in the airways of MD-2 deficient mice [60], all of which suggests functional complementation of Der p 2 and MD-2. A recent study by Arlian *et al*., [62] showed that HDM extracts activated human dermal endothelial cells to express adhesion molecules and to produce a range of cytokines. The authors demonstrated that although this response was partially due to contaminating LPS in the mite extracts, these effects could not be wholly attributed to this ligand and therefore other molecules (allergens for example) in the mite extracts were also involved. A similar result was also described by Hammad et al., [63]. We recently confirmed the findings of previous studies which identified the presence of a *P. ovis* homologue of Der p 2 (termed Pso o 2) which, like Der p 2, contains a conserved MD-2-related lipid-recognition (ML) domain and has 41% amino acid identity with Der p 2 [11], [64], [65]. Pso o 2 also maintains a number of important features deemed to be necessary for MD-2 like activity, including six conserved cysteine residues, N-glycosylation sites and conserved lysine residues involved in binding LPS [60], [61], [66]. Previous studies in mice have shown that low levels of LPS exposure can bias towards the development of a Th2-type immune response [67]. Sheep scab infestations occur in an environment that is highly likely to be contaminated with LPS, either from host commensal bacteria on the skin or from mite commensal/ symbiotic bacteria [68]. As such it is conceivable that LPS is present at the site of infestation and, in conjunction with mite-derived allergens like Pso o 2, could trigger TLR4, instigating a pro-inflammatory response. The involvement of Pso o 2 in the enhancement of TLR4-signalling is currently under investigation.

### Conclusions

This study has further described the temporal nature of the host transcriptional response to sheep scab infestation and has identified a key role for the transcriptional regulator NF-kB in the instigation of the ensuing pro-inflammatory response. The analysis of the temporal cascades of NF-kB-regulated gene expression revealed distinct patterns of transcriptional control involved in the production of the major mediators of the inflammatory response, including an array of cytokines, chemokines, selectins and integrins. One of the most prominent signs of sheep scab infestation is the rapid inflammation that occurs on the skin surface, with reddening of the skin visible within 1 hour of exposure. Due to the rapidity of this response it is clear that it must be instigated through initial interactions between the mites/mite factors and the host keratinocytes in the outer layer of skin, the stratum corneum. The identification of an LPS-type response during early *P. ovis* infestation leads to the intriguing possibility that a mite allergen, Pso o 2, may act as a potential mediator of this response through enhancement of host TLR4-signalling. These findings may prove to be crucial in the identification and design of novel methods of disease control for this economically important parasite.

## Materials and Methods

### Microarray study and statistical analysis

The details of the transcriptomic analysis of ovine skin following infestation with *P. ovis* have been reported previously [11]. Differential gene expression across the time course of infestation was determined using a one way-analysis of variance (ANOVA) with a Student-Newman-Keuls (SNK) post-hoc test in Genespring GX 11.0 (Agilent Technologies, UK) comparing each of the 5 time points, non-infected (time  = 0), 1, 3, 6 and 24 hours post infestation (hpi) across all animals. Multiple test correction was performed using the Benjamini & Hochberg False Discovery Rate (FDR) procedure with an FDR corrected p-value cut-off of ≤0.05 and a fold change cut-off of ≥2.0 [156].

### Transcription factor binding site analysis

Analysis of transcription factor binding site enrichment across the genes from the individual expression clusters was performed with the oPOSSUM Human Single Site Analaysis package [12]. This application is designed to detect over-represented conserved single sites in sets of co-expressed genes by comparison against a pre-compiled background set of genes [12]. The analysis was performed using the Human orthologous promoter sequence for each ovine gene searching 5000 bp upstream and 2000bp downstream of the defined transcription start site (TSS) and with the vertebrate taxonomic group selected. Otherwise the default settings were applied, using the most stringent level of conservation which equated to a minimum of 70%. A matrix match threshold value of 80% was applied, which represents the percentage match between the optimal canonical transcription factor binding motif and the matched site in the promoter. In all cases the top 20 results were selected for further analysis and a Z-score of greater than 10 and a Fisher Score of ≤0.01 applied, these parameters being considered as an accurate measure of statistical significance [12].

### Clustering of gene expression data

Clustering was performed in Genespring GX 11.0 (Agilent, UK) based on both time (hpi) and individual entities (genes in the relevant dataset). Clustering was carried out using the hierarchical clustering option with a Pearson centered distance metric.

### Pathway analysis

Data were analysed with Ingenuity Pathways Analysis (IPA) (Ingenuity Systems,www.ingenuity.com). Gene clusters identified from the time course analysis and clustering described formed the input data set. Each gene identifier was mapped to its corresponding gene object in Ingenuity's Knowledge Base. Gene networks were then algorithmically generated based on their connectivity and assigned a score (a numerical value used to rank networks according to how relevant they are to the genes in the input dataset). IPA uses a right-tailed Fisher's test to calculate the p-value for networks. A score of 10 indicates a p = 10^−10^ chance that genes in that network are associated solely by chance. A network is a graphical representation of the molecular relationships between molecules, molecules being represented as nodes and relationship between nodes being represented as edges. All edges are supported by at least 1 reference from the literature. Networks were analysed to identify the biological functions and/or diseases most significant to the genes in that network. Canonical pathway analysis identified the biological pathways of most significance. The significance of the association between the data set and canonical pathway was determined based on two parameters: (1) Ratio of the number of genes from the data set that map to the pathway divided by the total number of genes that map to the canonical pathway and (2) p-value calculated using Fisher's exact test determining the probability that the association between the genes in the data set and the canonical pathway is due to chance alone.

## Supporting Information

File S1 **Detailed description of the 151 putative transcription factor genes from the original list of 1,383 (with annotation available) differentially expressed in ovine skin following infestation with** ***P. ovis*** **.**
(DOCX)

File S2 **List of 799 genes with known or predicted NF-kB binding motifs within the 5**′ **promoter region.** This list of 799 genes was constructed by combining lists of genes with known or predicted NF-kB binding motifs within their promoter regions from 3 separate sources: The online resource NF-kB.org (http://www.nf-kb.org) and from studies by Tian *et al*., [37] and Schreiber *et al*., [36].
(TXT)

File S3 **List of 259 genes from the original list of 1,383 genes differentially expressed following infestation of skin with** ***P. ovis*** **with known or predicted NF-kB binding motifs within the 5**′ **promoter region.** This list describes the 259 genes with known or predicted NF-kB binding motifs within the 5′ promoter region that were also differentially expressed in sheep skin following infestation with *P. ovis*.
(TXT)
