# Supplementary material for: Host Transcription Factors in the Immediate Pro-Inflammatory Response to the Parasitic Mite Psoroptes ovis
Source: PLoS One. 2011 Sep 7;6(9):e24402. doi: 10.1371/journal.pone.0024402 (PMC3168495; doi:10.1371/journal.pone.0024402)
Supplement: File S1 — Detailed description of the 151 putative transcription factor genes from the original list of 1,383 (with annotation available) differentially expressed in ovine skin following infestation with P. ovis . (DOCX) [file pone.0024402.s001.docx]

| **Gene Symbol** | **Entrez Gene Name** | **Temporal cluster** | **Peak expression (hpi)** | **Molecule Type** |
| --- | --- | --- | --- | --- |
| SPI1 | spleen focus forming virus (SFFV) proviral integration oncogene spi1 | 4 | 24 | transcription regulator |
| IRF8 | interferon regulatory factor 8 | 4 | 24 | transcription regulator |
| ILF3 | interleukin enhancer binding factor 3, 90kDa | 4 | 24 | transcription regulator |
| ILF3 | interleukin enhancer binding factor 3, 90kDa | 4 | 24 | transcription regulator |
| IRF8 | interferon regulatory factor 8 | 4 | 24 | transcription regulator |
| SP140 | SP140 nuclear body protein | 4 | 24 | transcription regulator |
| TRIM6 | tripartite motif-containing 6 | 4 | 24 | other |
| HCLS1 | hematopoietic cell-specific Lyn substrate 1 | 4 | 24 | transcription regulator |
| TRIM6-TRIM34 |  | 4 | 24 |  |
| ELK3 | ELK3, ETS-domain protein (SRF accessory protein 2) | 4 | 24 | transcription regulator |
| SP100 | SP100 nuclear antigen | 4 | 24 | transcription regulator |
| ENO1 | enolase 1, (alpha) | 4 | 24 | transcription regulator |
| CALR | calreticulin | 4 | 24 | transcription regulator |
| IRF9 | interferon regulatory factor 9 | 4 | 24 | transcription regulator |
| ENO1 | enolase 1, (alpha) | 4 | 24 | transcription regulator |
| EFEMP2 | EGF-containing fibulin-like extracellular matrix protein 2 | 4 | 24 | other |
| IRF5 | interferon regulatory factor 5 | 4 | 24 | transcription regulator |
| CEBPD | CCAAT/enhancer binding protein (C/EBP), delta | 3 | 6 | transcription regulator |
| BCL2A1 | BCL2-related protein A1 | 3 | 6 | other |
| AHR | aryl hydrocarbon receptor | 3 | 6 | ligand-dependent nuclear receptor |
| HIF1A | hypoxia inducible factor 1, alpha subunit (basic helix-loop-helix transcription factor) | 3 | 6 | transcription regulator |
| HIF1A | hypoxia inducible factor 1, alpha subunit (basic helix-loop-helix transcription factor) | 3 | 6 | transcription regulator |
| POLD3 | polymerase (DNA-directed), delta 3, accessory subunit | 3 | 6 | transcription regulator |
| HIF1A | hypoxia inducible factor 1, alpha subunit (basic helix-loop-helix transcription factor) | 3 | 6 | transcription regulator |
| LITAF | lipopolysaccharide-induced TNF factor | 3 | 6 | transcription regulator |
| AATF | apoptosis antagonizing transcription factor | 3 | 6 | transcription regulator |
| NMI | N-myc (and STAT) interactor | 3 | 6 | transcription regulator |
| STAT3 | signal transducer and activator of transcription 3 (acute-phase response factor) | 3 | 6 | transcription regulator |
| SP110 | SP110 nuclear body protein | 3 | 6 | other |
| IFI16 | interferon, gamma-inducible protein 16 | 3 | 6 | transcription regulator |
| AHR | aryl hydrocarbon receptor | 3 | 6 | ligand-dependent nuclear receptor |
| TAF9 | TAF9 RNA polymerase II, TATA box binding protein (TBP)-associated factor, 32kDa | 3 | 6 | transcription regulator |
| AATF | apoptosis antagonizing transcription factor | 3 | 6 | transcription regulator |
| AHR | aryl hydrocarbon receptor | 3 | 6 | ligand-dependent nuclear receptor |
| BATF | basic leucine zipper transcription factor, ATF-like | 3 | 6 | transcription regulator |
| RCL1 | RNA terminal phosphate cyclase-like 1 | 3 | 6 | enzyme |
| TEAD4 | TEA domain family member 4 | 3 | 6 | transcription regulator |
| TFB2M | transcription factor B2, mitochondrial | 3 | 6 | enzyme |
| RCL1 | RNA terminal phosphate cyclase-like 1 | 3 | 6 | enzyme |
| E2F4 | E2F transcription factor 4, p107/p130-binding | 3 | 6 | transcription regulator |
| MYBBP1A | MYB binding protein (P160) 1a | 3 | 6 | transcription regulator |
| XBP1 | X-box binding protein 1 | 3 | 6 | transcription regulator |
| XBP1 | X-box binding protein 1 | 3 | 6 | transcription regulator |
| BATF3 | basic leucine zipper transcription factor, ATF-like 3 | 3 | 6 | transcription regulator |
| XBP1 | X-box binding protein 1 | 3 | 6 | transcription regulator |
| DAXX | death-domain associated protein | 3 | 6 | transcription regulator |
| S1PR3 | ankyrin repeat domain 1 (cardiac muscle) | 3 | 6 | transcription regulator |
| CREM | cAMP responsive element modulator | 2 | 3 | transcription regulator |
| CREM | cAMP responsive element modulator | 2 | 3 | transcription regulator |
| NFKBIZ | nuclear factor of kappa light polypeptide gene enhancer in B-cells inhibitor, zeta | 2 | 3 | transcription regulator |
| CREM | cAMP responsive element modulator | 2 | 3 | transcription regulator |
| RCAN1 | regulator of calcineurin 1 | 2 | 3 | transcription regulator |
| NFKBIA | nuclear factor of kappa light polypeptide gene enhancer in B-cells inhibitor, alpha | 2 | 3 | other |
| IRF1 | interferon regulatory factor 1 | 2 | 3 | transcription regulator |
| ETS2 | v-ets erythroblastosis virus E26 oncogene homolog 2 (avian) | 2 | 3 | transcription regulator |
| MYC | v-myc myelocytomatosis viral oncogene homolog (avian) | 2 | 3 | transcription regulator |
| MYC | v-myc myelocytomatosis viral oncogene homolog (avian) | 2 | 3 | transcription regulator |
| NFKBIE | nuclear factor of kappa light polypeptide gene enhancer in B-cells inhibitor, epsilon | 2 | 3 | transcription regulator |
| ELF3 | E74-like factor 3 (ets domain transcription factor, epithelial-specific ) | 2 | 3 | transcription regulator |
| ETS1 | v-ets erythroblastosis virus E26 oncogene homolog 1 (avian) | 2 | 3 | transcription regulator |
| ELL2 | elongation factor, RNA polymerase II, 2 | 2 | 3 | transcription regulator |
| NFKB2 | nuclear factor of kappa light polypeptide gene enhancer in B-cells 2 (p49/p100) | 2 | 3 | transcription regulator |
| RELB | v-rel reticuloendotheliosis viral oncogene homolog B | 2 | 3 | transcription regulator |
| HIF1A | hypoxia inducible factor 1, alpha subunit (basic helix-loop-helix transcription factor) | 2 | 3 | transcription regulator |
| BCL3 | B-cell CLL/lymphoma 3 | 2 | 3 | transcription regulator |
| RUNX1 | runt-related transcription factor 1 | 2 | 3 | transcription regulator |
| SPI1 | spleen focus forming virus (SFFV) proviral integration oncogene spi1 | 2 | 3 | transcription regulator |
| NFKBIB | nuclear factor of kappa light polypeptide gene enhancer in B-cells inhibitor, beta | 2 | 3 | transcription regulator |
| PRDM1 | PR domain containing 1, with ZNF domain | 2 | 3 | transcription regulator |
| PAF1 | Paf1, RNA polymerase II associated factor, homolog (S. cerevisiae) | 2 | 3 | other |
| IFNAR2 | interferon (alpha, beta and omega) receptor 2 | 2 | 3 | transmembrane receptor |
| TRIM10 | tripartite motif-containing 10 | 2 | 3 | other |
| MEOX1 | mesenchyme homeobox 1 | 2 | 3 | transcription regulator |
| IFNAR2 | interferon (alpha, beta and omega) receptor 2 | 2 | 3 | transmembrane receptor |
| BCL2L11 | BCL2-like 11 (apoptosis facilitator) | 2 | 3 | other |
| ETS1 | v-ets erythroblastosis virus E26 oncogene homolog 1 (avian) | 2 | 3 | transcription regulator |
| RUNX1 | runt-related transcription factor 1 | 2 | 3 | transcription regulator |
| ATRX | alpha thalassemia/mental retardation syndrome X-linked (RAD54 homolog, S. cerevisiae) | 2 | 3 | transcription regulator |
| NFKB1 | nuclear factor of kappa light polypeptide gene enhancer in B-cells 1 | 2 | 3 | transcription regulator |
| HIVEP2 | human immunodeficiency virus type I enhancer binding protein 2 | 2 | 3 | transcription regulator |
| ATF3 | activating transcription factor 3 | 1 | 1 | transcription regulator |
| BTG2 | BTG family, member 2 | 1 | 1 | transcription regulator |
| EGR1 | early growth response 1 | 1 | 1 | transcription regulator |
| ZFP36 | zinc finger protein 36, C3H type, homolog (mouse) | 1 | 1 | transcription regulator |
| EGR1 | early growth response 1 | 1 | 1 | transcription regulator |
| JUNB | jun B proto-oncogene | 1 | 1 | transcription regulator |
| JUNB | jun B proto-oncogene | 1 | 1 | transcription regulator |
| EGR1 | early growth response 1 | 1 | 1 | transcription regulator |
| EGR1 | early growth response 1 | 1 | 1 | transcription regulator |
| FOS | v-fos FBJ murine osteosarcoma viral oncogene homolog | 1 | 1 | transcription regulator |
| NR4A1 | nuclear receptor subfamily 4, group A, member 1 | 1 | 1 | ligand-dependent nuclear receptor |
| FOS | v-fos FBJ murine osteosarcoma viral oncogene homolog | 1 | 1 | transcription regulator |
| FOS | v-fos FBJ murine osteosarcoma viral oncogene homolog | 1 | 1 | transcription regulator |
| EGR1 | early growth response 1 | 1 | 1 | transcription regulator |
| FOS | v-fos FBJ murine osteosarcoma viral oncogene homolog | 1 | 1 | transcription regulator |
| EGR3 | early growth response 3 | 1 | 1 | transcription regulator |
| HEY1 | hairy/enhancer-of-split related with YRPW motif 1 | 1 | 1 | transcription regulator |
| NFIB | nuclear factor I/B | 1 | 1 | transcription regulator |
| JUND | jun D proto-oncogene | 1 | 1 | transcription regulator |
| PER1 | period homolog 1 (Drosophila) | 1 | 1 | other |
| JUN | jun oncogene | 1 | 1 | transcription regulator |
| MEF2C | myocyte enhancer factor 2C | 1 | 1 | transcription regulator |
| PRRX1 | paired related homeobox 1 | 1 | 1 | transcription regulator |
| PPARG | peroxisome proliferator-activated receptor gamma | 1 | 1 | ligand-dependent nuclear receptor |
| NR3C2 | nuclear receptor subfamily 3, group C, member 2 | 1 | 1 | ligand-dependent nuclear receptor |
| EGR1 | early growth response 1 | 1 | 1 | transcription regulator |
| NOSTRIN | nitric oxide synthase trafficker | 1 | 1 | transcription regulator |
| NR3C2 | nuclear receptor subfamily 3, group C, member 2 | 1 | 1 | ligand-dependent nuclear receptor |
| JUN | jun oncogene | 1 | 1 | transcription regulator |
| JUN | jun oncogene | 1 | 1 | transcription regulator |
| IFI17 | interferon, gamma-inducible protein 17 | 1 | 1 | transcription regulator |
| HOXC6 | homeobox C6 | 6 | -3 | transcription regulator |
| LMO2 | LIM domain only 2 (rhombotin-like 1) | 6 | -3 | other |
| NR2F2 | nuclear receptor subfamily 2, group F, member 2 | 6 | -3 | ligand-dependent nuclear receptor |
| MEF2C | myocyte enhancer factor 2C | 7 | -6 | transcription regulator |
| ZNF652 | zinc finger protein 652 | 7 | -6 | other |
| KLF11 | Kruppel-like factor 11 | 7 | -6 | transcription regulator |
| ETV1 | ets variant 1 | 7 | -6 | transcription regulator |
| PBX1 | pre-B-cell leukemia homeobox 1 | 7 | -6 | transcription regulator |
| TRIM2 | tripartite motif-containing 2 | 7 | -6 | other |
| GATA6 | GATA binding protein 6 | 7 | -6 | transcription regulator |
| TRIM2 | tripartite motif-containing 2 | 7 | -6 | other |
| DBP | D site of albumin promoter (albumin D-box) binding protein | 7 | -6 | transcription regulator |
| NFIB | nuclear factor I/B | 7 | -6 | transcription regulator |
| BCL11A | B-cell CLL/lymphoma 11A (zinc finger protein) | 7 | -6 | transcription regulator |
| NFIB | nuclear factor I/B | 7 | -6 | transcription regulator |
| TSC22D3 | TSC22 domain family, member 3 | 7 | -6 | transcription regulator |
| NFIA | nuclear factor I/A | 7 | -6 | transcription regulator |
| SMARCA2 | SWI/SNF related, matrix associated, actin dependent regulator of chromatin, subfamily a, member 2 | 7 | -6 | transcription regulator |
| TSC22D3 | TSC22 domain family, member 3 | 7 | -6 | transcription regulator |
| NFIA | nuclear factor I/A | 7 | -6 | transcription regulator |
| TCEA3 | transcription elongation factor A (SII), 3 | 7 | -6 | transcription regulator |
| IRF2BP2 | interferon regulatory factor 2 binding protein 2 | 7 | -6 | transcription regulator |
| ZNF277 | zinc finger protein 277 | 7 | -6 | transcription regulator |
| BARX2 | BARX homeobox 2 | 7 | -6 | transcription regulator |
| MYCN | v-myc myelocytomatosis viral related oncogene, neuroblastoma derived (avian) | 7 | -6 | transcription regulator |
| HOXD4 | homeobox D4 | 7 | -6 | transcription regulator |
| ESR1 | estrogen receptor 1 | 7 | -6 | ligand-dependent nuclear receptor |
| NR2F1 | nuclear receptor subfamily 2, group F, member 1 | 7 | -6 | ligand-dependent nuclear receptor |
| JUN | jun oncogene | 8 | -24 | transcription regulator |
| KLF4 | Kruppel-like factor 4 (gut) | 8 | -24 | transcription regulator |
| KLF9 | Kruppel-like factor 9 | 8 | -24 | transcription regulator |
| ZNF187 | zinc finger protein 187 | 8 | -24 | transcription regulator |
| ELF5 | E74-like factor 5 (ets domain transcription factor) | 8 | -24 | transcription regulator |
| ID4 | inhibitor of DNA binding 4, dominant negative helix-loop-helix protein | 8 | -24 | transcription regulator |
| LEF1 | lymphoid enhancer-binding factor 1 | 8 | -24 | transcription regulator |
| CRTC1 | CREB regulated transcription coactivator 1 | 8 | -24 | transcription regulator |
| LEF1 | lymphoid enhancer-binding factor 1 | 8 | -24 | transcription regulator |
| HOXC13 | homeobox C13 | 8 | -24 | transcription regulator |
| HOXC13 | homeobox C13 | 8 | -24 | transcription regulator |

**Supplementary File S1.** Detailed description of the 151 putative transcription factor genes from the original list of 1,383 (with annotation available) differentially expressed in ovine skin following infestation with *P. ovis*.
